# Anorexia nervosa and bulimia nervosa: a Mendelian randomization study of gut microbiota

**DOI:** 10.3389/fmicb.2024.1396932

**Published:** 2024-05-09

**Authors:** Zongliang Yu, Manping Guo, Binyang Yu, Yiming Wang, Zian Yan, Rui Gao

**Affiliations:** ^1^Graduate School, Beijing University of Chinese Medicine, Beijing, China; ^2^Xiyuan Hospital, China Academy of Chinese Medical Sciences, Beijing, China; ^3^Postdoctoral Research Station, China Academy of Chinese Medical Sciences, Beijing, China; ^4^Postdoctoral Works Station, Yabao Pharmaceutical Group Co., Ltd., Yuncheng, China

**Keywords:** anorexia nervosa, bulimia nervosa, gut microbiota, Mendelian randomization analysis, eating disorders

## Abstract

**Background:**

Anorexia nervosa (AN) and bulimia nervosa (BN) poses a significant challenge to global public health. Despite extensive research, conclusive evidence regarding the association between gut microbes and the risk of AN and BN remains elusive. Mendelian randomization (MR) methods offer a promising avenue for elucidating potential causal relationships.

**Materials and methods:**

Genome-wide association studies (GWAS) datasets of AN and BN were retrieved from the OpenGWAS database for analysis. Independent single nucleotide polymorphisms closely associated with 196 gut bacterial taxa from the MiBioGen consortium were identified as instrumental variables. MR analysis was conducted utilizing R software, with outlier exclusion performed using the MR-PRESSO method. Causal effect estimation was undertaken employing four methods, including Inverse variance weighted. Sensitivity analysis, heterogeneity analysis, horizontal multivariate analysis, and assessment of causal directionality were carried out to assess the robustness of the findings.

**Results:**

A total of 196 bacterial taxa spanning six taxonomic levels were subjected to analysis. Nine taxa demonstrating potential causal relationships with AN were identified. Among these, five taxa, including *Peptostreptococcaceae*, were implicated as exerting a causal effect on AN risk, while four taxa, including *Gammaproteobacteria*, were associated with a reduced risk of AN. Similarly, nine taxa exhibiting potential causal relationships with BN were identified. Of these, six taxa, including *Clostridiales*, were identified as risk factors for increased BN risk, while three taxa, including *Oxalobacteraceae*, were deemed protective factors. *Lachnospiraceae* emerged as a common influence on both AN and BN, albeit with opposing effects. No evidence of heterogeneity or horizontal pleiotropy was detected for significant estimates.

**Conclusion:**

Through MR analysis, we revealed the potential causal role of 18 intestinal bacterial taxa in AN and BN, including *Lachnospiraceae*. It provides new insights into the mechanistic basis and intervention targets of gut microbiota-mediated AN and BN.

## 1 Introduction

Recent epidemiological studies indicate that 16% of global mortality can be attributed to mental disorders, rendering them among the leading causes of disability and premature death worldwide (Murray et al., 2020; Arias et al., 2022). Eating disorders are one of the common types of mental disorders, exhibiting an age-standardized prevalence of 174.0 per 100,000 population. The prevalence of eating disorders in high-income countries is about three times the global average (GBD 2019 Mental Disorders Collaborators, 2022). Anorexia nervosa (AN) and bulimia nervosa (BN) represent the most prevalent types of eating disorders and are uniquely identified as psychiatric conditions with elevated mortality risk in the Global Burden of Disease Study 2019 (GBD 2019 Demographics Collaborators, 2020). A common feature of patients with both disorders is an excessive focus on weight and body shape and attempts to control weight. Patients with AN often adopt excessive behaviors to avoid weight gain, while patients with BN experience multiple overeating followed by inappropriate weight-compensating behaviors (Timko et al., 2019). Studies have suggested that AN and BN may share the same psychopathological features and have reciprocal transformations, manifesting as similar behaviors, such as impulsivity and compulsion (Howard et al., 2020).

Cognitive behavioral therapy is the first-line treatment for adults with AN and BN (Treasure et al., 2020). Nevertheless, a meta-analysis found that over 60% of patients failed to fully abstain from core symptoms even after receiving the best available treatments (Slade et al., 2018). Maudsley model and focal psychodynamic psychotherapy are also first-line treatments for adults with AN. For adults with BN, third-wave behavioral therapies are feasible attempts, such as dialectical behavior therapy, acceptance and commitment therapy. However, previous evaluations have shown little difference in efficacy between these therapies and cognitive behavioral therapy (Byrne et al., 2017; Linardon et al., 2017). For adolescents with AN and BN, family-based interventions are recommended as first-line treatments by international evidence-based guidelines (Hilbert et al., 2017). However, a Cochrane review suggests that the evidence favoring family-based interventions over standard treatment or other psychological approaches is not very solid (Fisher et al., 2019). Therefore, new treatments for AN and BN, such as the novel ghrelin receptor agonist, and transcranial direct-current stimulation are still worthy of in-depth research and exploration (van Passel et al., 2020; Solmi et al., 2021).

The gut microbiota intricately participates in various physiological processes crucial for human well-being, including metabolic regulation and immune homeostasis (Fujisaka et al., 2018; Chen et al., 2022). Emerging evidence suggests that the microbiota and the central nervous system communicate bidirectionally via the microbiota-gut-brain axis, thereby influencing the pathophysiology of psychiatric disorders (Agirman et al., 2021; Cryan and Mazmanian, 2022; Fan et al., 2023). A recent study found that, multiple bacterial taxa, such as *Clostridium*, were altered in AN and correlated with estimates of eating behavior and mental health (Fan et al., 2023). A systematic review provided a clearer picture of the gut microbiota characteristics of AN patients. Preserved alpha-diversity and decreased beta-diversity were found in the qualitative synthesis. Three gut microbes (*Alistipes, Parabacterioides and Roseburia*), are able to effectively differentiate patients from controls (Di Lodovico et al., 2021). Analogously, investigations into BN have corroborated a potential association between gut microbiota and BN development. Different pathological behaviors may be associated with a reduction in microbial diversity and typical microbiota-derived metabolites (Castellini et al., 2023). These findings underscore the intertwined relationship between gut microbiota and the progression of AN and BN. However, the causal link between specific bacterial taxa and AN and BN necessitates further elucidation.

Conventionally, randomized controlled trials represent the gold standard for establishing causality. However, ethical dilemmas, compliance issues and difficulties in family coordination add additional challenges to the study of AN and BN, especially for adolescents (Frostad and Bentz, 2022; Tsiandoulas et al., 2023). Mendelian randomization (MR) offers a pragmatic alternative approach for assessing causality by utilizing single nucleotide polymorphisms (SNPs) as genetic instrumental variables (IVs) to statistically infer the causal impact of exposures on outcomes (Kurilshikov et al., 2021). This method emulates the random allocation process, thereby mitigating the influence of confounding variables (Emdin et al., 2017). Moreover, since the microbiome does not induce alterations in an individual’s DNA sequence, analyzing the causal relationship between gut microbiota and AN and BN through MR studies holds practical clinical relevance (Thomas, 2019). In this investigation, we employed two-sample MR analysis to scrutinize the potential causal role of gut microbiota in AN and BN, delineated specific pathogenic bacterial taxa, and elucidated their similarities, disparities, and associated mechanisms.

## 2 Materials and methods

### 2.1 Study overview

According to the law of independent assortment, genetic variants will be randomly assorted to gametes during meiosis. MR analysis uses this law as a principle to simulate randomized controlled trials using SNPs as genetic IVs. Because of this, MR study is seen as an appropriate method for analyzing the causal effects of exposure on clinical outcomes.

**Figure 1:**
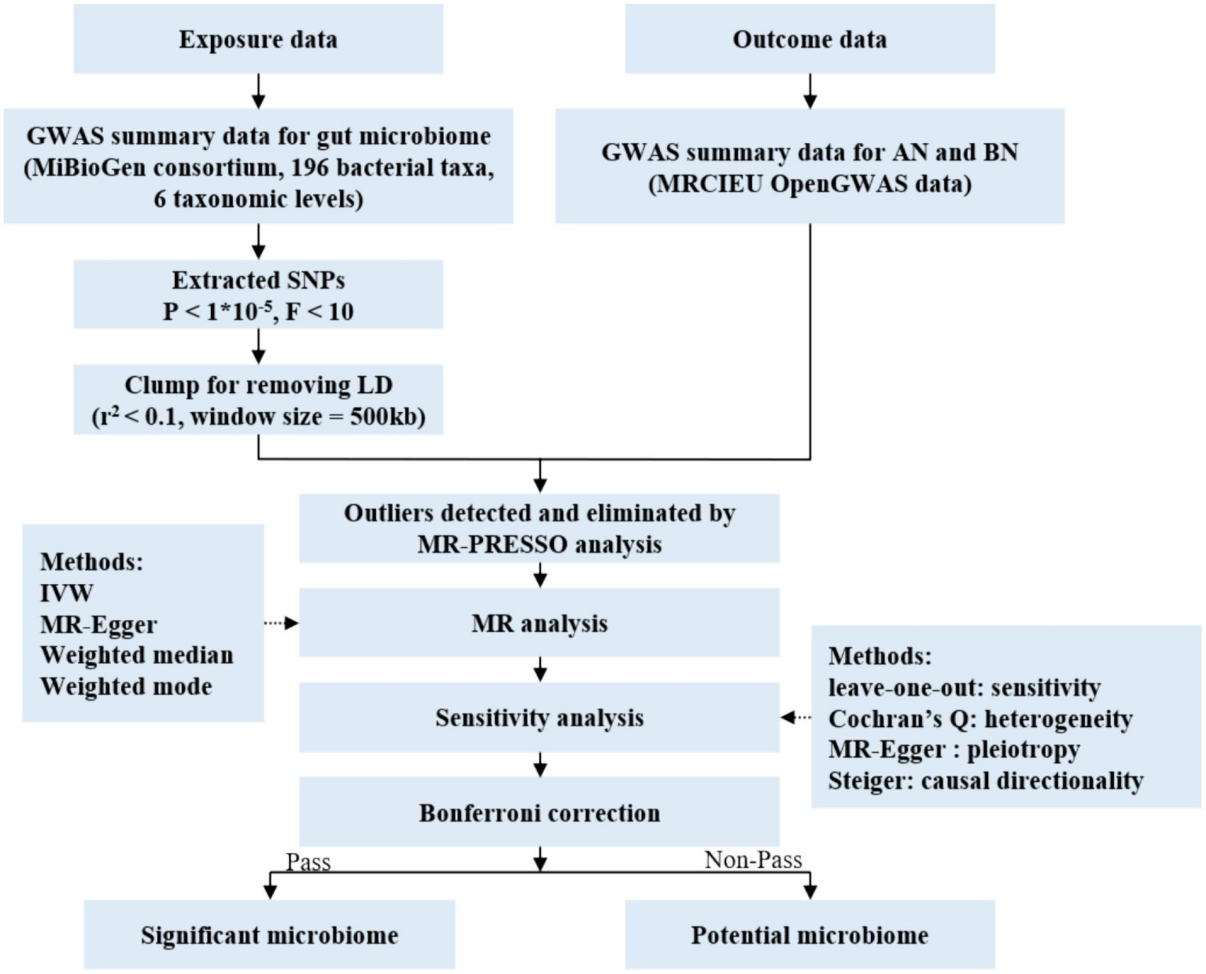
Flow chart of the study. GWAS, genome wide association study; SNPs, single nucleotide polymorphisms; MR, Mendelian randomization; LD, linkage disequilibrium; IVW, inverse variance weighted.

In this study, each bacterial taxon contained in the gut microbiota was categorized as a separate exposure. Of all, 196 gut microbiota taxa were selected as exposures (Kurilshikov et al., 2021), AN and BN were defined as the outcome variable (Wade et al., 2013; Duncan et al., 2017). Two-sample MR analysis was conducted using summary statistics from genome-wide association studies (GWAS) to discern which bacterial taxa were causally associated with AN and BN, respectively. The overall design of the study is shown in Figure 1.

The analysis adhered to three pivotal assumptions inherent to MR studies (Emdin et al., 2017; Skrivankova et al., 2021): (1) genetic variation in exposed populations correlates with the exposure of interest, (2) genetic variation remains independent of confounding variables, and (3) genetic variation influences outcomes solely through the exposure of interest. The causal relationship between exposure factors and outcome factors was confirmed by testing 3 hypotheses that confirmed an indirect relationship between IV and outcome variables and was achieved only by exposure factors. A detailed information chart is provided in Supplementary material (Skrivankova et al., 2021).

### 2.2 Genetic association data screening for AN and BN

All SNP data related to AN and BN were sourced from the MRC IEU OpenGWAS data infrastructure (Lyon et al., 2021), developed by the MRC Integrated Epidemiology Unit at the University of Bristol. This repository aggregates genetic associations from 50,037 GWAS datasets, totaling 346,312,366,530 genetic associations. Considering sample size, sequencing depth, ethnicity, and data recency, GWAS datasets for AN and BN authored by Duncan et al. (2017) and Wade et al. (2013), respectively, were selected. These studies encompassed 13 study cohorts with a combined sample size of 16,919 individuals, drawn from diverse pedigrees to mitigate bias (Supplementary Tables S1, S2).

### 2.3 The selection of IVs

The genetic IVs of each bacterial taxon were obtained from the MiBioGen consortium, which comprised 18,340 individuals from 24 cohorts. The consortium utilized standardized analytical pipelines for both microbiota phenotype and genotype, ensuring uniform data processing methods. This approach was employed to mitigate potential variations introduced by technical differences in generating microbiota data (Kurilshikov et al., 2021). This study used three different regions (V4: 10,413 samples, 13 cohorts, V3–V4: 4,211 samples, 6 cohorts and V1–V2: 3,716 samples, 5 cohorts) (V: hypervariable region sequencing for identifying bacterial taxa) of the 16S rRNA gene to analysis the composition of gut microbiota and identified genetic variants that influent the relative abundance of microbial taxa by use of microbiota Quantitative Trait loci mapping (Kurilshikov et al., 2021). Following the removal of 15 unknown bacterial taxa, the final dataset encompassed 196 taxa (Supplementary Table S3).

SNP screening entailed the following criteria (Sanna et al., 2019): (1) genome-wide SNPs with significance (*p* < 1 × 10^−5^), (2) exclusion of weak IVs with an *F*-statistic <10, (3) linkage disequilibrium (LD) testing to ensure independence of selected IVs (*r*^2^ < 0.1 within a 500 kb range), and (4) removal of SNPs with incompatible or palindromic allele frequencies.

### 2.4 MR analysis and sensitivity assessment

MR analysis was conducted using R software (version 4.3.2) to evaluate the potential causal effect of gut microbes on AN and BN risk. MR estimates for each SNP were derived using the Wald ratio method, with meta-analysis performed using four methods: Inverse variance weighted (IVW), MR Egger, weighted median, and weighted mode (Hartwig et al., 2017). The IVW method, recognized for its superior accuracy under practical conditions (Bowden et al., 2016), served as the primary analytical approach, supplemented by the other three methods. Bonferroni correction was applied at each taxonomic level (phylum, class, order, family, and genus) to account for multiple comparisons. MR estimates with *p* < 0.05 were considered statistically significant, while false discovery rate (FDR) *p*-values <0.05 denoted unequivocal significance. All estimates were expressed as odds ratio (OR) with a 95% confidence interval (CI) per standard deviation increase in the corresponding exposure.

The MR-PRESSO test was employed to identify outliers and address heterogeneity. In instances of detected heterogeneity among genetic IVs, outliers were eliminated, and MR analysis was re-executed. Sensitivity analyses were conducted via the leave-one-out method, Cochran’s *Q* statistic was utilized to assess potential heterogeneity, and the MR Egger intercept test was employed to estimate horizontal pleiotropy. Finally, the Steiger method facilitated causal directionality analysis to mitigate potential effects of reverse causation.

## 3 Results

### 3.1 Overview of genetic IVs

Multiple SNPs were considered for each of the 196 bacterial taxa, following stringent screening based on genome-wide significance thresholds, LD testing, and validation of the *F* statistic. Any SNPs identified as outliers by MR-PRESSO (global test: *p* < 0.05) were excluded. All retained SNPs exhibited *F*-statistics exceeding 10, indicating a robust correlation between the genetic IVs and the corresponding bacterial taxa. The final roster of retained SNPs and pertinent statistics are detailed in Supplementary Tables S4, S5.

### 3.2 Relationship of intestinal bacterial taxa to AN and BN

Among the 196 taxon phenotypes examined, stringent Bonferroni correction did not reveal any unequivocal and significant causal relationships between gut microbiome and the risk of AN or BN (FDR *p* < 0.05). Nevertheless, based on analyses employing the IVW method and three additional methods, we ascertained potential causal relationships between certain intestinal taxa and the risk of AN or BN (*p* < 0.05).

As shown in Table 1, we identified nine taxa with potential causal relationships with AN. Among these nine taxa, *Peptostreptococcaceae* (IVW OR = 1.341, 95% CI 1.039–1.733, *p* = 0.024), *Coprococcus3* (IVW OR = 1.563, 95% CI 1.084–2.252, *p* = 0.017), *Escherichia Shigella* (IVW OR = 1.490, 95% CI 1.109–2.003, *p* = 0.008), *Lachnospiraceae NC2004 group* (IVW OR = 1.287, 95% CI 1.019–1.626, *p* = 0.034), *Lachnospiraceae UCG010* (IVW OR = 1.363, 95% CI 1.018–1.825, *p* = 0.038) were determined to have a causal effect on AN risk. And *Cyanobacteria* (IVW OR = 0.724, 95% CI 0.563–0.930, *p* = 0.012), *Gammaproteobacteria* (IVW OR = 0.619, 95% CI 0.406–0.943, *p* = 0.026), *Mollicutes RF9* (IVW OR = 0.740, 95% CI 0.584–0.939, *p* = 0.013), and *Eubacterium brachy group* (IVW OR = 0.778, 95% CI 0.643–0.942, *p* = 0.010) trended toward a lower risk of AN. Among the other three MR analysis methods (MR Egger, weighted median, weighted mode), only the weighted median method for the *Coprococcus3* and *Lachnospiraceae NC2004 group* and the MR Egger calculations for *Escherichia Shigella* were statistically significant. It is noteworthy that all these results are in agreement with the IVW calculation method for this group, which to some extent reflects the stability of the results.

**Table 1:** Significant MR results of potential causal effect of gut microbiota on AN.

Level	Exposure	*N* SNPs	Method	OR	95% CI	*p*-value
Phylum	*Cyanobacteria*	8	IVW	0.724	0.563–0.930	0.012^*^
			MR Egger	1.142	0.479–2.719	0.775
			Weighted median	0.767	0.541–1.088	0.137
			Weighted mode	0.545	0.299–0.994	0.088
Class	*Gammaproteobacteria*	6	IVW	0.619	0.406–0.943	0.026^*^
			MR Egger	0.533	0.141–2.011	0.406
			Weighted median	0.645	0.376–1.108	0.113
			Weighted mode	0.626	0.310–1.265	0.249
Order	*Mollicutes RF9*	13	IVW	0.740	0.584–0.939	0.013^*^
			MR Egger	0.776	0.381–1.580	0.498
			Weighted median	0.760	0.549–1.053	0.099
			Weighted mode	0.773	0.481–1.242	0.308
Family	*Peptostreptococcaceae*	14	IVW	1.341	1.039–1.733	0.024^*^
			MR Egger	1.436	0.805–2.561	0.244
			Weighted median	1.288	0.914–1.814	0.148
			Weighted mode	1.212	0.730–2.011	0.47
Genus	*Coprococcus3*	9	IVW	1.563	1.084–2.252	0.017^*^
			MR Egger	3.927	1.072–14.381	0.078
			Weighted median	1.704	1.036–2.804	0.036^*^
			Weighted mode	1.902	0.868–4.168	0.147
	*Escherichia Shigella*	10	IVW	1.490	1.109–2.003	0.008^*^
			MR Egger	2.772	1.184–6.491	0.047^*^
			Weighted median	1.384	0.924–2.072	0.115
			Weighted mode	1.158	0.559–2.401	0.702
	*Eubacterium brachy group*	9	IVW	0.778	0.643–0.942	0.010^*^
			MR Egger	0.829	0.346–1.985	0.686
			Weighted median	0.827	0.642–1.065	0.141
			Weighted mode	0.861	0.599–1.239	0.444
	*Lachnospiraceae NC2004 group*	9	IVW	1.287	1.019–1.626	0.034^*^
			MR Egger	0.956	0.359–2.547	0.931
			Weighted median	1.461	1.057–2.019	0.022^*^
			Weighted mode	1.598	0.965–2.644	0.106
	*Lachnospiraceae UCG010*	10	IVW	1.363	1.018–1.825	0.038^*^
			MR Egger	1.879	0.881–4.005	0.141
			Weighted median	1.256	0.858–1.839	0.240
			Weighted mode	1.180	0.697–1.996	0.553

^*^Represents that *p* meets the significance threshold. *N* SNPs represents the number of SNPs being used as IVs; OR, odds ratio; CI, confidence interval; IVW, inverse variance weighted, MR, Mendelian randomization.

**Figure 2:**
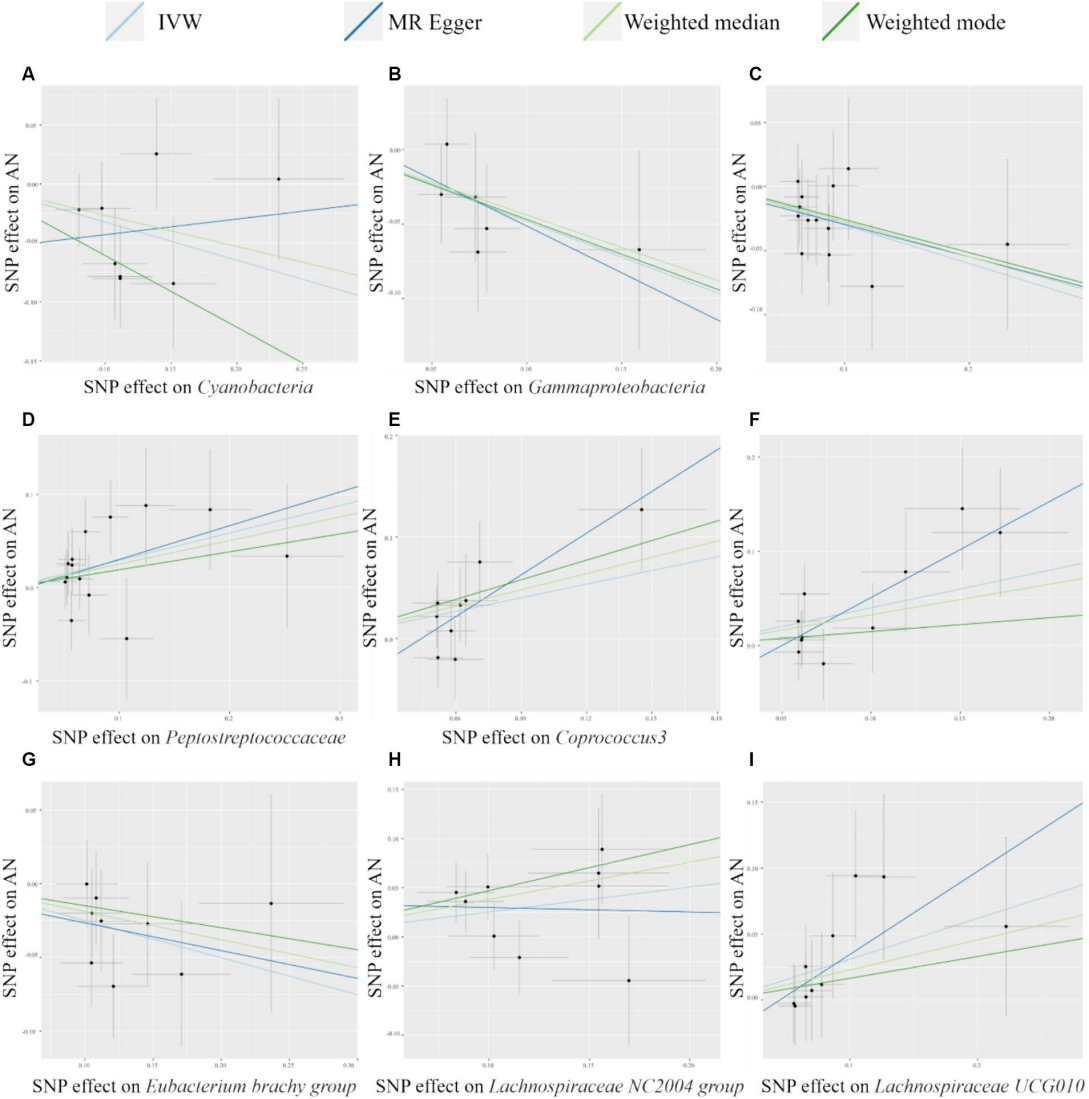
Scatter plots of MR analysis of potential causal effect of 9 gut microbiota on AN. **(A)** *Cyanobacteria*, **(B)** *Gammaproteobacteria*, **(C)** *Mollicutes RF9*, **(D)** *Peptostreptococcaceae*, **(E)** *Coprococcus3*, **(F)** *Escherichia Shigella*, **(G)** *Eubacterium brachy group*, **(H)** *Lachnospiraceae NC2004 group*, **(I)** *Lachnospiraceae UCG010*. IVW, inverse variance weighted; AN, anorexia nervosa; MR, Mendelian randomization; SNP, single nucleotide polymorphism.

The scatter plot (Figure 2) visualized the causal relationship between gut bacterial taxa and AN. Each point in the graph represents a SNP, and the short lines of the cross at each point reflect its 95% CI. The abscissa is the effect of the SNP on the exposure (gut microbe), and the ordinate is the effect of the SNP on the outcome (AN). The slash lines of different colors represent the MR fitting results of different calculation methods. A slope greater than 0 indicates that the exposure factor (gut microbe) is a disadvantage of AN. For the fitting results of different methods, the results of IVW are generally the main ones. As shown in Figure 2, except for *Cyanobacteria* and *Lachnospiraceae NC2004 group*, the results of the other MR analysis methods for the remaining seven taxa were in agreement with their respective IVW results. However, despite the inconsistencies between the MR Egger method results of these two gut microbes and the IVW method, the results of the IVW method remained robust due to their wide CIs and loss of statistical significance.

**Figure 3:**
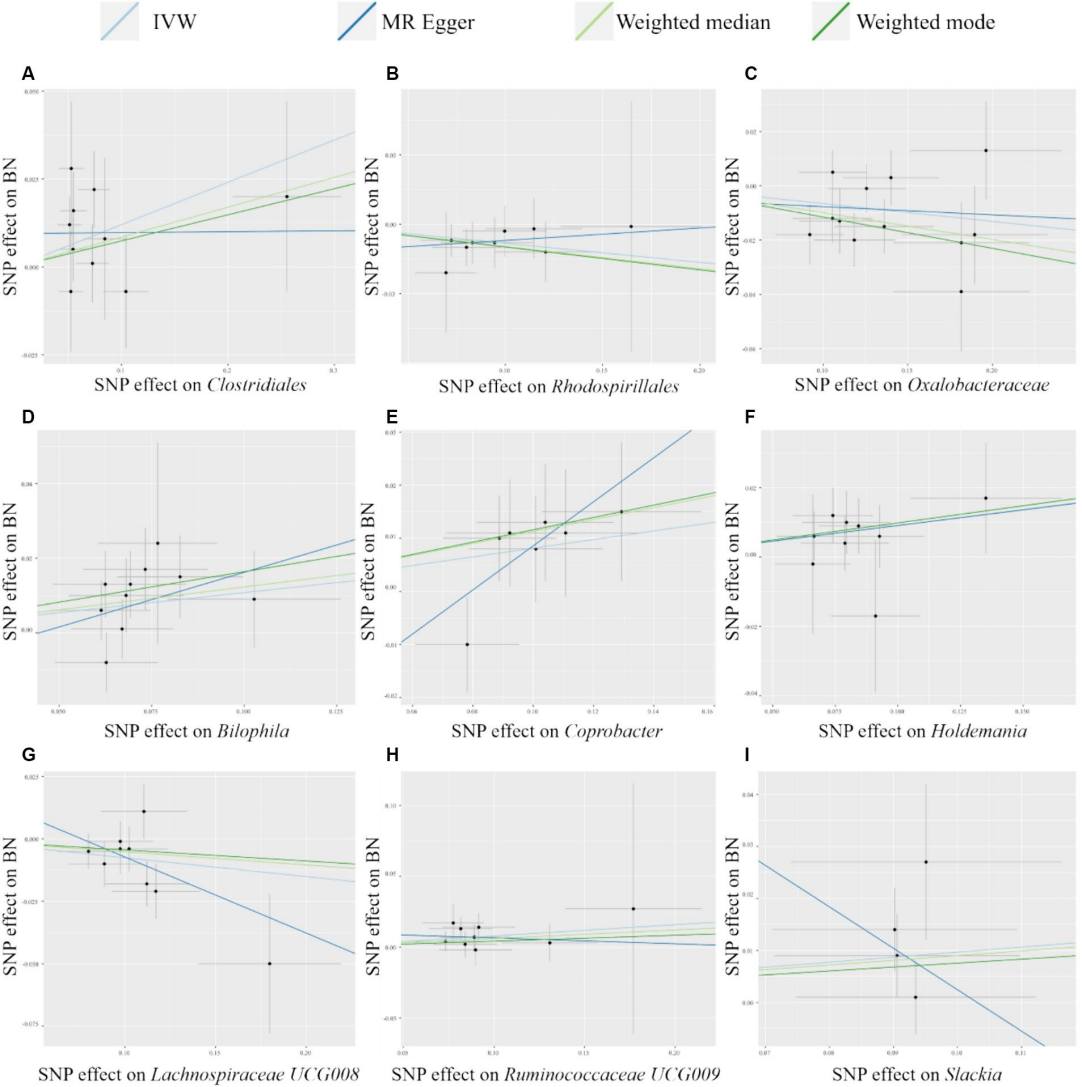
Scatter plots of MR analysis of potential causal effect of 9 gut microbiota on BN. **(A)** *Clostridiales*, **(B)** *Rhodospirillales*, **(C)** *Oxalobacteraceae*, **(D)** *Bilophila*, **(E)** *Coprobacter*, **(F)** *Holdemania*, **(G)** *Lachnospiraceae UCG008*, **(H)** *Ruminococcaceae UCG009*, **(I)** *Slackia*. IVW, inverse variance weighted; BN, bulimia nervosa; MR, Mendelian randomization; SNP, single nucleotide polymorphism.

As shown in Table 2, we identified nine taxa with potential causal relationships with BN. Among these nine taxa, *Clostridiales* (IVW OR = 1.128, 95% CI 1.016–1.252, *p* = 0.024), *Bilophila* (IVW OR = 1.114, 95% CI 1.021–1.214, *p* = 0.015), *Coprobacter* (IVW OR = 1.085, 95% CI 1.005–1.171, *p* = 0.037), *Holdemania* (IVW OR = 1.096, 95% CI 1.016–1.182, *p* = 0.018), *Ruminococcaceae UCG009* (IVW OR = 1.082, 95% CI 1.004–1.166, *p* = 0.038), and *Slackia* (IVW OR = 1.101, 95% CI 1.004–1.208, *p* = 0.041) were determined to be the increased risk of BN. While *Rhodospirillales* (IVW OR = 0.921, 95% CI 0.851–0.996, p = 0.038), *Oxalobacteraceae* (IVW OR = 0.937, 95% CI 0.890–0.985, *p* = 0.011), *Lachnospiraceae UCG008* (IVW OR = 0.927, 95% CI 0.871–0.987, *p* = 0.018) were identified as protective factors. Of the other three MR analysis methods, only the weighted median method for the *Rhodospirillales*, *Oxalobacteraceae*, *Bilophila*, and *Holdemania* were statistically significant. All of these results are consistent with the IVW calculations for this group, reflecting the stability of the study results. As shown in Figure 3, except for *Rhodospirillales*, *Ruminococcaceae UCG009*, and *Slackia*, the results of other MR analysis methods for the remaining six taxa were in agreement with their respective IVW results. However, the results of the IVW method remained robust due to the wide CIs and loss of statistical significance of the MR Egger method. It is worth noting that due to the relative shortage of SNPs in *Slackia*, the MR Egger method calculates too wide CIs, which to some extent reflects the disadvantage of the method being overly conservative.

**Table 2:** Significant MR results of potential causal effect of gut microbiota on BN.

Level	Exposure	*N* SNPs	Method	OR	95% CI	*p*-value
Order	*Clostridiales*	10	IVW	1.128	1.016–1.252	0.024^*^
			MR Egger	1.003	0.784–1.282	0.984
			Weighted median	1.089	0.941–1.259	0.252
			Weighted mode	1.077	0.900–1.288	0.438
	*Rhodospirillales*	9	IVW	0.921	0.851–0.996	0.038^*^
			MR Egger	1.055	0.687–1.620	0.813
			Weighted median	0.908	0.825–1.000	0.049^*^
			Weighted mode	0.906	0.806–1.019	0.137
Family	*Oxalobacteraceae*	12	IVW	0.937	0.890–0.985	0.011^*^
			MR Egger	0.970	0.757–1.244	0.817
			Weighted median	0.906	0.844–0.972	0.006^*^
			Weighted mode	0.891	0.788–1.007	0.092
Genus	*Bilophila*	10	IVW	1.114	1.021–1.214	0.015^*^
			MR Egger	1.341	0.735–2.447	0.367
			Weighted median	1.131	1.005–1.272	0.041^*^
			Weighted mode	1.178	0.989–1.403	0.100
	*Coprobacter*	7	IVW	1.085	1.005–1.171	0.037^*^
			MR Egger	1.514	0.891–2.573	0.185
			Weighted median	1.119	1.017–1.233	0.022^*^
			Weighted mode	1.123	0.986–1.279	0.130
	*Holdemania*	9	IVW	1.096	1.016–1.182	0.018^*^
			MR Egger	1.094	0.699–1.712	0.705
			Weighted median	1.103	1.002–1.215	0.045^*^
			Weighted mode	1.104	0.986–1.236	0.125
	*Lachnospiraceae UCG008*	9	IVW	0.927	0.871–0.987	0.018^*^
			MR Egger	0.738	0.491–1.108	0.186
			Weighted median	0.949	0.874–1.032	0.220
			Weighted mode	0.957	0.845–1.083	0.505
	*Ruminococcaceae UCG009*	9	IVW	1.082	1.004–1.166	0.038^*^
			MR Egger	0.960	0.602–1.531	0.867
			Weighted median	1.062	0.962–1.172	0.232
			Weighted mode	1.043	0.925–1.175	0.513
	*Slackia*	4	IVW	1.101	1.004–1.208	0.041^*^
			MR Egger	0.451	0.001–163.645	0.816
			Weighted median	1.094	0.973–1.231	0.135
			Weighted mode	1.079	0.942–1.234	0.353

^*^Represents that *p* meets the significance threshold. *N* SNPs represents the number of SNPs being used as IVs; OR, odds ratio; CI, confidence interval; IVW, inverse variance weighted, MR, Mendelian randomization.

### 3.3 Sensitivity analysis of MR results

Prior to conducting MR analysis, MR-PRESSO analysis was performed to exclude potential abnormal SNPs. Subsequent tests were undertaken to ensure the sensitivity of the findings and mitigate potential biases. The list of SNPs retained after the exclusion of aberrant SNPs by the MR-PRESSO method, along with relevant statistics, are presented in Supplementary Tables S4, S5. Further MR-PRESSO global test analysis revealed no outliers among IVs with significant causal relationships with AN or BN, as detailed in Table 3.

**Table 3:** Significance levels of different tests for MR results.

Outcome	Expose	Egger	*Q*	Steiger	MR-PRESSO global test
AN	*Cyanobacteria*	0.324	0.445	0.000	0.452
AN	*Gammaproteobacteria*	0.829	0.765	0.000	0.875
AN	*Mollicutes RF9*	0.894	0.959	0.000	0.980
AN	*Peptostreptococcaceae*	0.802	0.694	0.000	0.778
AN	*Coprococcus3*	0.190	0.764	0.000	0.651
AN	*Escherichia Shigella*	0.166	0.791	0.000	0.666
AN	*Eubacterium brachy group*	0.889	0.809	0.000	0.883
AN	*Lachnospiraceae NC2004 group*	0.559	0.541	0.000	0.629
AN	*Lachnospiraceae UCG010*	0.394	0.914	0.000	0.917
BN	*Clostridiales*	0.330	0.682	0.000	0.698
BN	*Rhodospirillales*	0.546	0.997	0.000	0.924
BN	*Oxalobacteraceae*	0.780	0.292	0.000	0.395
BN	*Bilophila*	0.558	0.655	0.000	0.088
BN	*Coprobacter*	0.268	0.805	0.000	0.745
BN	*Holdemania*	0.996	0.938	0.000	0.981
BN	*Lachnospiraceae UCG008*	0.301	0.434	0.000	0.473
BN	*Ruminococcaceae UCG009*	0.625	0.892	0.000	0.916
BN	*Slackia*	0.794	0.220	0.000	0.322

AN, anorexia nervosa; BN, bulimia nervosa; MR, Mendelian randomization.

However, there might be heterogeneity in IVs from different analysis platforms, experiments, and populations, which can affect the results of MR analysis (Cui et al., 2023). To assess potential heterogeneity, Cochran’s *Q* statistic was computed, with all *p*-values exceeding 0.05, indicating the absence of heterogeneity in the study. This finding was corroborated by the results of funnel plot analyses (Supplementary Figures S1, S3). When IVs affect outcomes through factors other than exposure factors, they indicate that IVs are pleioty. Pleiotropy leads to the failure of the independence and exclusivity assumptions (Bowden et al., 2015; Verbanck et al., 2018). MR Egger intercept test results yielded *p*-values greater than 0.05, signifying the absence of pleiotropy in the study. Similarly, Steiger method analyses affirmed that all significant findings implicated gut microbiota in AN or BN, with no evidence of reverse causality interference. Finally, sensitivity analysis employing the leave-one-out method demonstrated that the exclusion of any individual SNP did not substantially alter the results (Supplementary Figures S2, S4). These comprehensive analyses bolster the sensitivity and validity of the MR findings regarding the causal relationship between intestinal bacterial taxa and AN or BN.

## 4 Discussion

The escalating health and social burden attributed to eating disorders underscores the urgent need for effective treatments (Murray et al., 2020; Arias et al., 2022). It has been well-documented that eating disorders such as AN and BN interact with a range of mental and organic disorders (AlHadi et al., 2022). Blocking the progression of AN and BN by modulating the gut microbiota and its metabolites has received widespread attention from clinical researchers (Jiao et al., 2023; Himmerich and Treasure, 2024). However, the ethical dilemma and compliance problems in adolescent treatment add additional challenges to clinical research (Frostad and Bentz, 2022; Tsiandoulas et al., 2023). By leveraging genetic IVs, MR analysis facilitates a deeper understanding of the intricate interplay between gut microbiota and eating disorders, thereby offering promising avenues for targeted therapeutic interventions (Thomas, 2019).

Employing a two-sample MR analysis framework with GWAS datasets, we identified nine bacterial taxa with potential causal associations with AN and nine bacterial taxa with potential causal associations with BN. A previous study found that core microbiota depletion signs were observed in patients with AN. Overrepresented taxa in patients taxonomically belonged to *Alistipes*, *Clostridiales*, *Christensenellaceae*, and *Ruminococcaceae*. And underrepresented taxa were *Faecalibacterium*, *Agathobacter*, *Bacteroides*, *Blautia*, and *Lachnospira* (Prochazkova et al., 2021). Similar studies have found that gut microbiota-associated metabolites, such as trimethylamine N-oxide (Wagner-Skacel et al., 2022), choline (Doose et al., 2023), serotonin (Hata et al., 2019), and p-cresyl sulfate (Miyata et al., 2021), in the onset and progression of AN and BN.

In this investigation, we identified five bacterial taxa, including *Peptostreptococcaceae*, *Coprococcus3*, *Escherichia Shigella*, *Lachnospiraceae NC2004 group*, and *Lachnospiraceae UCG010*, as potential causes for the increased risk of AN. The findings concerning *Coprococcus* and *Escherichia Shigella* align with those reported in a previous meta-analysis study (Zang et al., 2023). Hanachi et al. (2019) reported a decrease in the richness and diversity of gut microbiota in severely malnourished AN patients who received enteral nutrition. This study further identified a negative correlation between the severity of functional intestinal disorders in AN patients and *Peptostreptococcaceae* by 16S rRNA analysis. This seems to contradict our conclusion that *Peptostreptococcaceae* is a potential cause of AN. However, the abundance of *Peptostreptococcaceae* is influenced by a variety of factors. The abundance of *Peptostreptococcaceae* is increased in stool samples from patients with ulcerative colitis and colorectal cancer (Cheng et al., 2020). And it tends to decrease after probiotic consumption, suggesting that the abundance of *Peptostreptococcaceae* is easily influenced by food intake and probiotics (Hibberd et al., 2017; Zaccaria et al., 2023). This appears to explain the decrease in the abundance of *Peptostreptococcaceae* in patients with functional intestinal disorders of AN after receiving enteral nutrition, which is not related to the onset of AN. This finding underscores the intricate role of the intestinal microbiota in the interplay between AN pathogenesis and its accompanying symptoms.

Our investigation also revealed that four bacterial taxa, namely *Cyanobacteria*, *Gammaproteobacteria*, *Mollicutes RF9*, and *Eubacterium brachy group*, exhibited the potential to reduce the risk of AN. Previous 16S rRNA investigations have highlighted correlations between *Eubacterium* and AN (Hanachi et al., 2019; Yuan et al., 2022), but directional causality remains elusive, a view that is confirmed by our findings. These microbes are one of the major producers of butyrate and play an important role in immunomodulatory processes at the intestinal mucosal level, which may be one reason for the reduced risk in AN patients (Yuan et al., 2022). *Cyanobacteria* are single-celled prokaryotes capable of oxygen-producing photosynthesis, which are mainly used in fields such as fertilizers and fuels (Arias et al., 2021). Recent studies have found that *Cyanobacteria* contains a variety of bioactive components that can induce autophagy and apoptosis, regulate epigenetic modifications, and exert antitumor effects (Bouyahya et al., 2024). Xu et al. (2019) found that *Cyanobacteria* were associated with neuropsychological behavior induced by long-term alcohol exposure, and the mechanism may be related to the secretion of brain-derived neurotrophic factor. Depression-like behavior induced by a high-fat diet in mice is also associated with increased abundance of *Cyanobacteria* (Hassan et al., 2019). Similarly, *Gammaproteobacteria* have been shown to be abundant in young adults with major depressive disorder, regardless of psychotropic medication (Liu et al., 2020). Mice exposed to chronic social defeat stress showed mild depressive-like behavior and an increase in the abundance of *Mollicutes* (Kosuge et al., 2021). And a study on anti-anxiety drugs found that (R)-ketamine might work by downregulating *Mollicutes* (Yang et al., 2017). Based on the available evidence, these gut microbes (*Cyanobacteria, Gammaproteobacteria, Mollicutes*) are all positively associated with depression, and their potential role in reducing AN risk remains to be confirmed by clinical studies. These three types of gut microbes seem to help distinguish between AN and depression, which have similar clinical manifestations.

In a previously published MR analysis, *Actinobacteria*, *Bilophila*, *Holdemania*, *Lactobacillus*, *Ruminococcaceae UCG009*, and two unknown gut microbes were identified as risk factors for the development of AN, which is very different from our conclusions (Xia et al., 2023). Interestingly, the results are highly similar to those of our BN study. Reviewing the original published GWAS datasets, we found that the main reason for this was because the earlier GWAS datasets could not be distinguished from the two disease subtypes at a technical level due to the crossover of AN and BN diagnoses (Boraska et al., 2014). At the same time, the limitations of detection techniques have resulted in relatively small numbers of SNPs captured from earlier datasets, limiting the exploration of more critical causal associations (Duncan et al., 2017). Therefore, our study can be regarded as an update of this study, which provides cross-validation of the findings of BN in this study.

In our results, six taxa, including *Clostridiales*, *Bilophila*, *Coprobacter*, *Holdemania*, *Ruminococcaceae UCG009*, and *Slackia*, are potential contributors to the increased risk of BN. *Ruminococcaceae* are positively associated with autism, depression, and are abundant in patients with AN (Prochazkova et al., 2021). Similar studies showed that the abundance of *Clostridiales* in feces of activity-based anorexia mice with food restriction increased significantly (Breton et al., 2019). Given the crossover nature and common pathological basis of AN and BN diagnoses, their potential risks to BN are promising. *Bilophila* is an opportunistic pathogen, and the close association of increased abundance with intestinal inflammation has been confirmed (Alexander et al., 2023; Zahavi et al., 2023). Recent studies have found that a ketogenic diet can exacerbate cognitive impairment caused by intermittent hypoxia, the mechanism of which is associated with impairment of hippocampal function due to the enrichment of gut microbes such as *Bilophila* (Olson et al., 2021). Changes in *Bilophila* abundance have been observed in patients with mental disorders such as autism spectrum disorders. As for *Coprobacter*, its abundance is currently thought to be associated with chronic insomnia and cognitive function (Feng et al., 2021). Similarly, *Holdemania* exhibits a high abundance in patients with depression (Barandouzi et al., 2020) and is closely associated with anxiety and Parkinson’s disease (Jang et al., 2020). *Slackia* stands out as a typical causative agent, however, recent studies suggest that it may be involved in the development of Alzheimer’s disease (Nagpal et al., 2019). Therefore, although there are no reports about *Coprobacter*, *Holdemania* and *Slackia* in patients with AN or BN, their research is still worthy of attention.

Our study identified three taxa—*Rhodospirillales*, *Oxalobacteraceae*, and *Lachnospiraceae UCG008*—as protective factors for BN. *Rhodospirillales* is mainly used in water purification and new energy sources (Chun et al., 2018). Zhang et al. (2023) found that sleep deprivation led to a significant increase in the abundance of *Rhodospirillales* and enhanced pro-inflammatory cytokine responses as well as learning and memory impairments in mice. Another study found a significant increase in the abundance of *Rhodospirillales* in socially isolated mice (Siddi et al., 2024). Studies of *Oxalobacteraceae* have found that it reduces the risk of delirium, attention deficit hyperactivity disorder (Wang et al., 2023; Yu et al., 2023). Therefore, these gut microbes may have a close relationship with mental disorders, and further clinical research on AN and BN is worth exploring.

It is notable that AN and BN share similar clinical mechanisms, yet previous studies have not consistently identified a specific group of gut microorganisms commonly associated with both disorders. In our current investigation, we observed that while the *Lachnospiraceae NC2004 group* and *Lachnospiraceae UCG010* were identified as risk factors for AN, *Lachnospiraceae UCG008* emerged as a protective factor for BN. Previous studies have found that *Lachnospiraceae NC2004 group* was able to reduce the risk of gastroduodenal ulcers, and was able to reduce circulating inflammatory cytokine levels (Xue et al., 2023). *Lachnospiraceae UCG010* may reduce the risk of cholelithiasis and narcolepsy (Liu et al., 2023; Sheng et al., 2024). In contrast, *Lachnospiraceae UCG008* has been shown to be associated with a higher risk of periodontitis (Ye et al., 2023), and is a potential risk factor for hemorrhagic stroke (Shen et al., 2023). Based on current evidence, we cannot definitively explain the contradiction between the different genera of *Lachnospiraceae*, but it appears to be related to inflammation and immunity (Sun et al., 2021; Zeng et al., 2023). However, despite belonging to distinct strains, *Lachnospiraceae* appears to be a common influencing factor for both AN and BN. Previous research has linked reductions in *Lachnospiraceae* to an increased risk of developing depression (Liu et al., 2022), autism (Li et al., 2023), and Alzheimer’s disease (Hung et al., 2022). The elevated risk of AN observed in our study further underscores the widespread association of *Lachnospiraceae* with mental disorders (Wang et al., 2023). Notably, although *Lachnospiraceae* have exhibited favorable organismal protective effects across a spectrum of diseases (McCulloch et al., 2022; Yan et al., 2023), their contrasting effects on AN and BN suggest a complex relationship between gut microbiota and disease that warrants further elucidation. The exact mechanism underlying these observations remains to be clarified through additional clinical intervention studies.

The therapeutic feasibility of microbial supplements for the treatment of psychiatric disorders has gained considerable recognition (Sanada et al., 2020; Góralczyk-Bińkowska et al., 2022). Liu et al. confirmed that probiotic supplementation mitigated intestinal damage induced by dietary restriction in AN patients (Liu et al., 2021). Subsequent clinical investigations substantiated that administering a probiotic complex reduces inflammation levels and gastrointestinal distress symptoms in AN patients (Gröbner et al., 2022). There is a potential causal relationship between the gut microbiota and AN and BN identified in this study, and the regulation of these microbiota by probiotics may be a promising therapeutic target for AN and BN.

In this study, we have, for the first time, elucidated the causal relationship of certain intestinal taxa with AN and BN, offering novel perspectives for subsequent mechanistic exploration and drug research. Leveraging causal inference through MR design effectively circumvented the influences of confounding bias and reverse causation. Furthermore, we conducted rigorous tests to ensure the sensitivity of our findings. However, this study does entail some limitations. Firstly, the sample size and the number of relevant loci in the current gut microbiota GWAS data are constrained. To mitigate the risk of inadequate IVs at the genus and species levels, potentially leading to imprecise bacterial characterization, we conducted summary analyses of bacterial features at higher taxonomic levels. It is anticipated that with advancements in microbiome GWAS and the accumulation of larger sample sizes, more specific bacterial features will be discerned and complemented. Secondly, to enhance statistical power, the gut microbiota and disease GWAS datasets analyzed in this study were amalgamated from multi-source samples. While this approach facilitates broader extrapolation of conclusions, it may introduce some heterogeneity in the results. Therefore, despite employing a meticulous methodology to exclude heterogeneous data, the results should be interpreted cautiously.

## 5 Conclusion

In summary, our study identified the potential causal involvement of 18 intestinal bacterial taxa, notably including *Lachnospiraceae*, in the pathogenesis of AN and BN through MR analysis. These potentially valuable gut microbiota may indicate the risk of disease development and provide feasible targets for the pathogenesis of AN and BN and probiotic therapy. Further clinical intervention and intestinal microbial testing have broad prospects for the research and treatment of AN and BN.

## Data availability statement

The original contributions presented in the study are included in the article/Supplementary material, further inquiries can be directed to the corresponding author.

## Author contributions

ZYu: Conceptualization, Data curation, Formal analysis, Investigation, Methodology, Writing – original draft. MG: Data curation, Formal analysis, Investigation, Writing – original draft. BY: Data curation, Formal analysis, Investigation, Writing – original draft. YW: Data curation, Investigation, Writing – original draft. ZYa: Investigation, Software, Writing – original draft. RG: Funding acquisition, Project administration, Resources, Supervision, Writing – review & editing.
