# Supplementary figures and images for: Anorexia nervosa and bulimia nervosa: a Mendelian randomization study of gut microbiota
Source: Front Microbiol. 2024 May 9;15:1396932. doi: 10.3389/fmicb.2024.1396932 (PMC11111991; doi:10.3389/fmicb.2024.1396932)

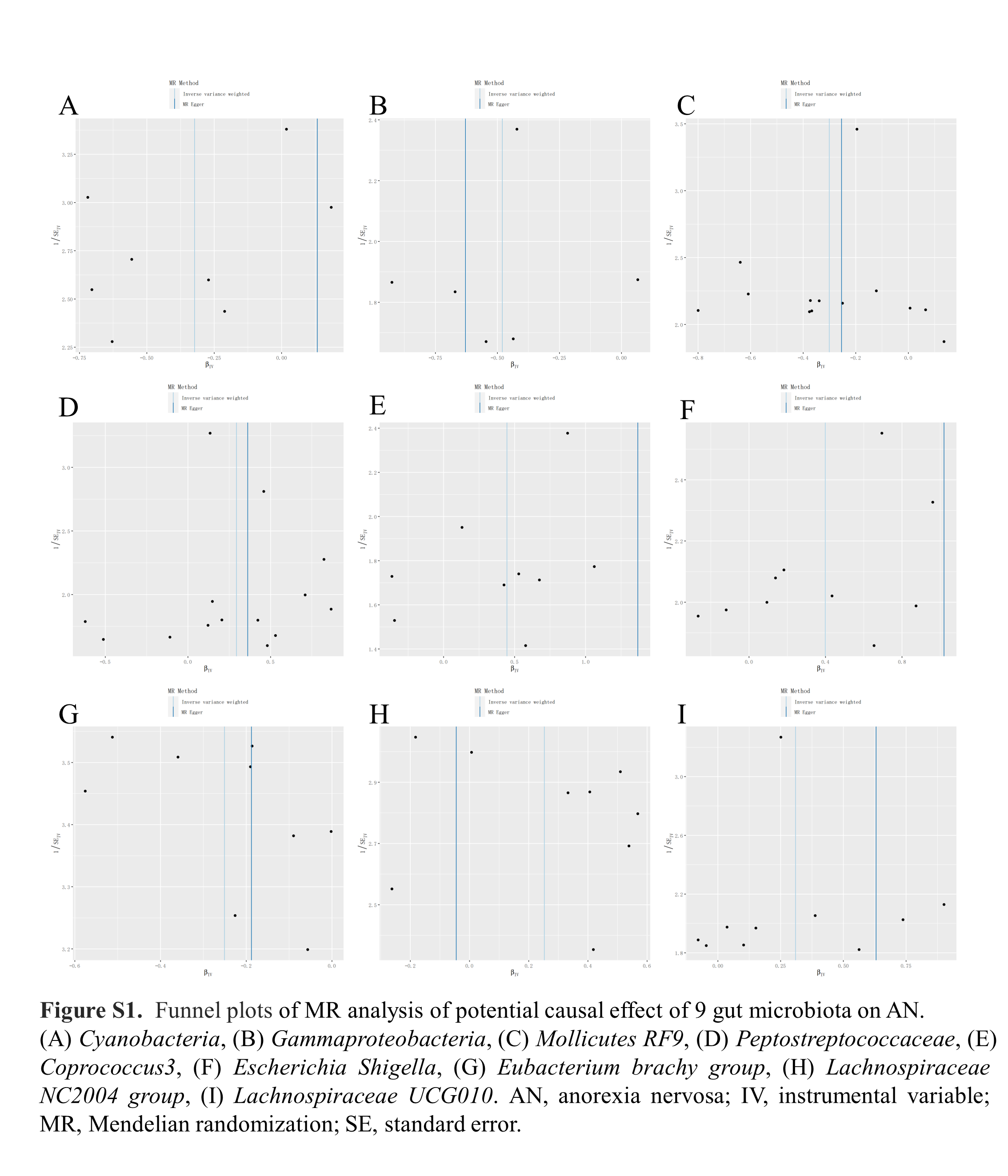

Supplement: Supplementary file 1 [file Image_1.TIF]

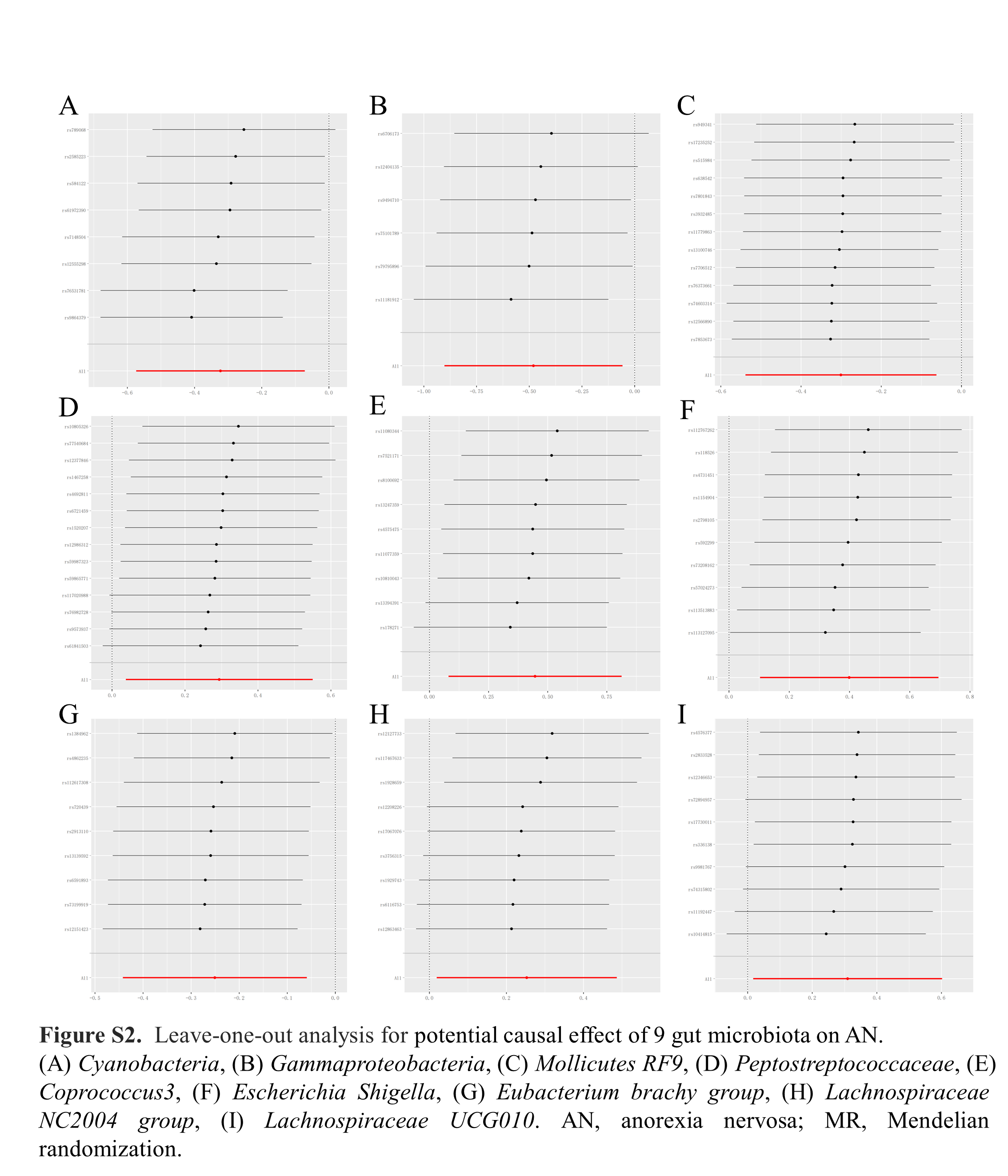

Supplement: Supplementary file 2 [file Image_2.TIF]

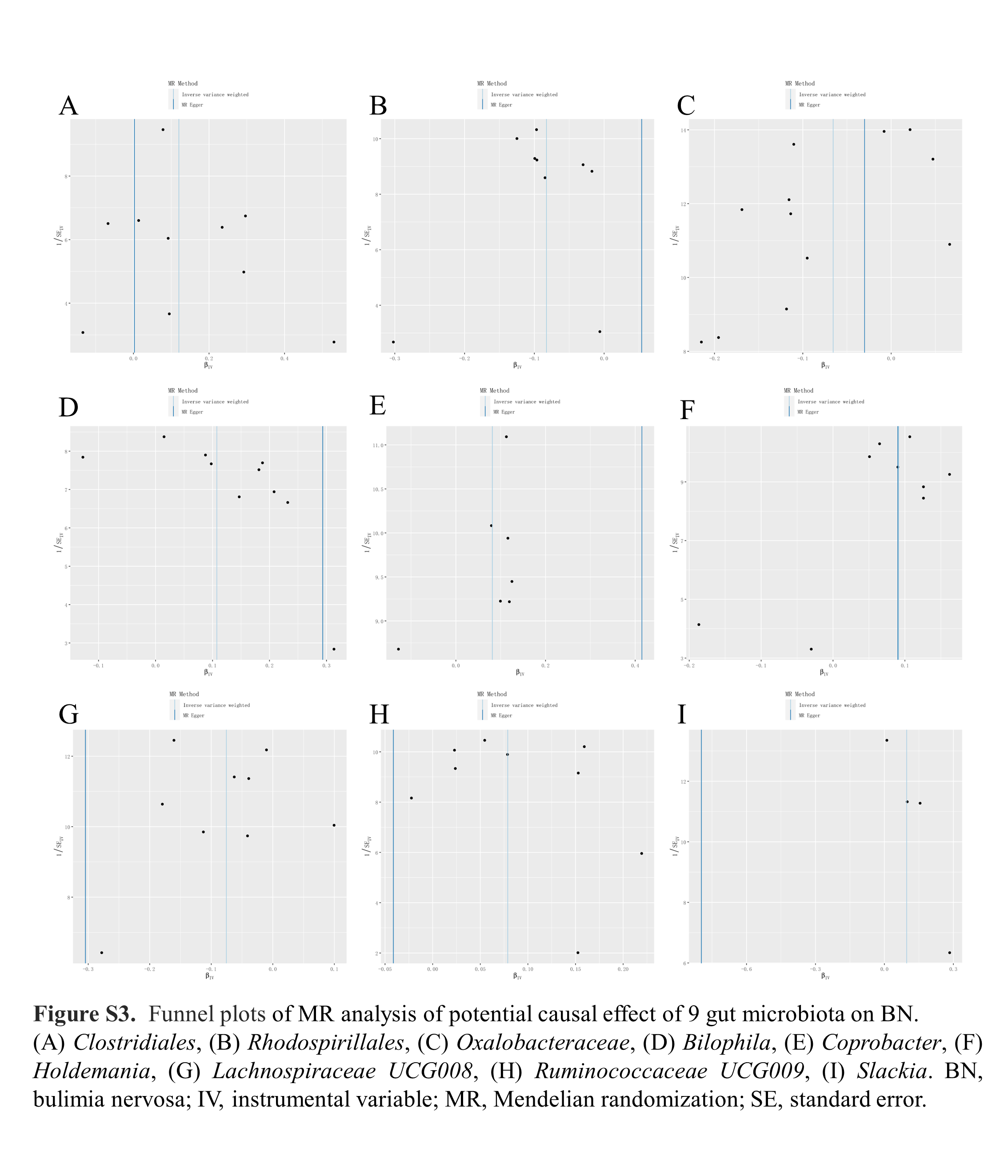

Supplement: Supplementary file 3 [file Image_3.TIF]

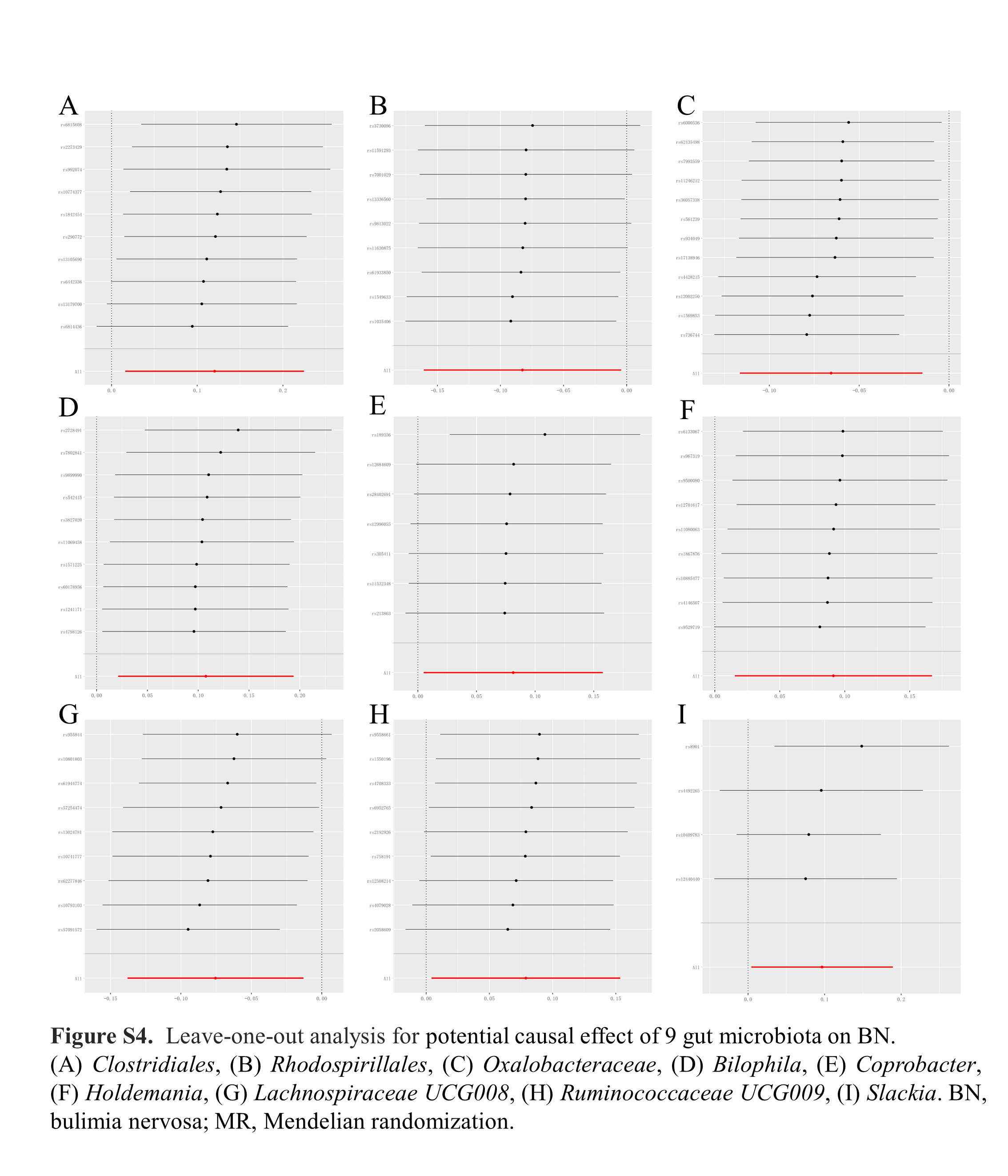

Supplement: Supplementary file 4 [file Image_4.TIF]
